# Effectiveness of the Lorodent Probiotic Lozenge in Reducing Plaque and *Streptococcus mutans* Levels in Orthodontic Patients: A Double-Blind Randomized Control Trial

**DOI:** 10.3389/froh.2022.884683

**Published:** 2022-04-27

**Authors:** Fatima Ebrahim, Sarah Malek, Kris James, Kyle MacDonald, Peter Cadieux, Jeremy Burton, Iacopo Cioffi, Celine Lévesque, Siew-Ging Gong

**Affiliations:** ^1^Faculty of Dentistry, University of Toronto, Toronto, ON, Canada; ^2^Departments of Surgery, Microbiology and Immunology, University of Western Ontario, London, ON, Canada

**Keywords:** plaque indices, lozenges, *Streptococcus mutans*, saliva, supragingival plaque, compliance

## Abstract

Orthodontic patients are at a significant risk for oral diseases due to increased plaque accumulation and oral bacterial dysbiosis. We aimed to determine the efficacy of the commercially available Lorodent Probiotic Complex at reducing plaque accumulation and *Streptococcus mutans* bacterial levels in adolescent orthodontic patients. Sixty adolescents undergoing fixed orthodontic treatment for a minimum of 6 months were recruited in a randomized, double-blind, parallel-group, placebo-controlled trial. They received either Lorodent probiotic lozenge (intervention, *n* = 30) or placebo lozenge (control, *n* = 30) orally every day for a 28-day administration period. Participants were assessed at four appointments (T1–T4) over a total of 56 days. Compliance and lozenge satisfaction were monitored. Saliva samples and supragingival plaques were collected for evaluation of *S. mutans* levels. Clinical assessment using a Plaque Index (PI) was used. Compliance with lozenge intake of all participants was over 90%. There was no significant change in the PI and composite PI scores in both placebo and probiotic groups at each time frame (all *p* > 0.05) or the relative *S. mutans* DNA levels in the saliva and plaque between the probiotic and placebo groups. The findings of high compliance and satisfaction with the probiotic lozenges combined with the study's rigorous design offer a baseline for subsequent testing of further potential probiotics (of varying formulations, concentrations), especially in adolescents.

## Introduction

A major negative effect of having fixed orthodontic appliances is a potential increase in oral diseases such as caries and periodontal diseases [1, 2]. Orthodontic patients can develop gingivitis [3] that may progress to periodontal disease [4]. Indeed, in children, gingivitis is the most commonly occurring periodontal disease [5] and approximately one third of North Americans suffer from either gingivitis or periodontal disease [6].

The formation of a dental biofilm, generally known as dental plaque, is fundamental to the disease processes observed in the oral cavity [7]. Dental plaque comprises an aggregation of bacteria, salivary components and their exopolymer matrix [8]. Substantial epidemiologic evidence has shown that the presence in plaque of aciduric and acidogenic bacteria such as *Streptococcus mutans* and lactobacilli plays an important role in the formation of caries [9]. Various preventive approaches against dental caries and periodontal diseases have been thoroughly researched (e.g., good oral hygiene practices, use of fluoride, sugar substitutes, remineralizing agents, and antimicrobial chemical rinses); in spite of this, the incidence of gingivitis and caries remains high in orthodontic patients [10]. There remains a need for a simple, adjunctive aid that can be used to reduce plaque accumulation and cariogenic oral pathogens, especially for at-risk patients such as the orthodontic population. Recent evidence suggests that probiotic therapy might be applied to the maintenance of oral health [11–13].

The World Health Organization defines probiotics as “live microorganisms which when administered in adequate amounts confer a health benefit on the host” [14]. The favorable effects of probiotic therapy are mainly achieved through the modulation of existing microbial flora associated with the host, thus attaining a balanced and healthy microbe-host relationship. Classic probiotic strains, such as those that belong to the genus *Lactobacillus*, have been tested for their ability to confer a probiotic effect in the oral cavity [15–19]. Indeed, probiotics have been used in orthodontics, with conflicting results [20–22]. The Lorodent Probiotic Complex (Integra Medical LLC) is a commercially available probiotic lozenge. It is a blend of six probiotic bacteria with *Streptococcus salivarius* BLIS K12 and five probiotic strains of the genus *Lactobacillus* (new nomenclature according to [23]): *Lacticaseibacillus paracasei* (previously *L. paracasei), Lactiplantibacillus plantarum* (previously *L. plantarum), Ligilactobacillus salivarius* (previously *L. salivarius*) *and Limosilactobacillus reuteri* (previously *L. reuteri*), and *Lactobacillus acidophilus*, being the key ingredients. The aim of the current study was to investigate the effectiveness of the Lorodent Probiotic Complex with a 3 × 10^5^ CFU/lozenge in reducing plaque and salivary/plaque *S. mutans* levels in adolescent participants undergoing fixed orthodontic appliance therapy. We hypothesized that the Lorodent Probiotic Complex lozenges could improve gingival health and reducing the level of *S. mutans* in plaque and saliva.

## Methods and Materials

### Trial Design

This randomized, double-blind, parallel-group, placebo-controlled trial was conducted at the Graduate Orthodontic Clinic at the University of Toronto, Faculty of Dentistry (Supplementary Material - CONSORT Checklist) and subjects were recruited between August 2014 to October 2014. The clinical trial was registered and conducted in compliance with Health Canada (#185428). The study was approved by the Research Ethics Board at the University of Toronto (protocol #30148). The study was registered at the University of Toronto Faculty of Dentistry Center for Clinical Research.

### Participants

Patients undergoing orthodontic treatment at the Graduate Orthodontic clinic, University of Toronto, Faculty of Dentistry, were screened by two orthodontic residents (FE and SH) under the supervision of an orthodontist (SGG) for eligibility by combining review of the medical and dental histories with a dental examination.

Eligibility criteria for the study included male and female subjects between 11 and 18 years of age, in healthy medical condition, who were not pregnant, not past or current users of alcohol or tobacco, and who had not used antimicrobial mouth rinses, probiotics, antibiotics or anti-inflammatory drugs within 1 month prior to the study.

#### Inclusion Criteria

- Fully erupted teeth #16, 21, 23, 36, 41, 43;
- No active caries;
- Mild to moderate plaque accumulation (Plaque Index [24] score of at least 1);
- Mild to moderate crowding;
- Undergoing fixed orthodontic treatment on both arches with edgewise metal orthodontic brackets on at least 20 teeth and 1st molars bonded for at least 6 months and submitted to a standardized orthodontic archwire sequence of 0.016″ NiTi, 0.016″ × 0.022″ NiTi, 0.019″ × 0.025″ NiTi, and 0.019″ × 0.025″ stainless steel.

#### Exclusion Criteria

- Allergies or sensitivity to milk or milk products, gluten, soy or any other ingredient present in the Lorodent Probiotic Complex;
- Existing dental caries or xerostomia;
- Any systemic condition that could directly affect gingival condition;
- Recent (within the past 45 days) or planned (within the next 90 days) surgery of any kind (major or minor);
- Participated in another clinical trial within 30 days prior to randomization;
- Experienced any nausea, fever, vomiting, bloody diarrhea or severe abdominal pain within the past 30 days;
- Patients with orthodontic bands.

Participants were randomly assigned to two groups, using a randomization protocol (details included in Supplementary Material—Methods and Materials). All participants received professional tooth cleaning at baseline, i.e., just before being enrolled in the clinical trial. The appliance was bonded in all participants using a standard protocol, e.g., standard bonding procedure using 37% phosphoric acid, Transbond™ Plus Self Etching primer (3M Unitek), and Transbond™ light cure adhesive (3M Unitek) (light cured Bis-GMA composite resin). Participants were withdrawn from the study for the following reasons: (1) personal reasons; (2) reports of fever, nausea, vomiting, diarrhea or severe abdominal pain after having used the probiotic; and (3) pregnancy, antibiotic use, or a severe medical condition. The clinical data and samples collected from such subjects were withdrawn from the study analysis.

### Interventions

The blueberry flavored Lorodent probiotic and placebo lozenges (Integra Medical Inc.) were chosen for this study because of its commercial availability and prior *in vitro* testing of its effectiveness against cariogenic bacteria by the company (Integra Medical, Inc.; data not shown). The probiotic complex was formulated to contain active probiotics (*S. salivarius* K12, and five probiotic strains of the genus *Lactobacillus*: *Lacticaseibacillus paracasei, Lactiplantibacillus plantarum, Lactobacillus acidophilus, Ligilactobacillus salivarius and Limosilactobacillus reuteri*) at a total probiotic concentration of ~3 × 10^5^ CFU/lozenge. In addition, both probiotic and placebo contained lactitol, inulin, dicalcium phosphate, blueberry flavor (natural), dextrose, fructose, stearic acid, citric acid, vanilla flavor (natural), and stevia rebaudiosidea (97%) as excipients. Stored in a −80°C freezer until distributed to participants, all subjects were instructed to store the lozenges in their fridge at home for the duration of the trial. All lozenges had expiration dates that exceeded the end of the trial study by a minimum of 6 months.

**Figure 1:**
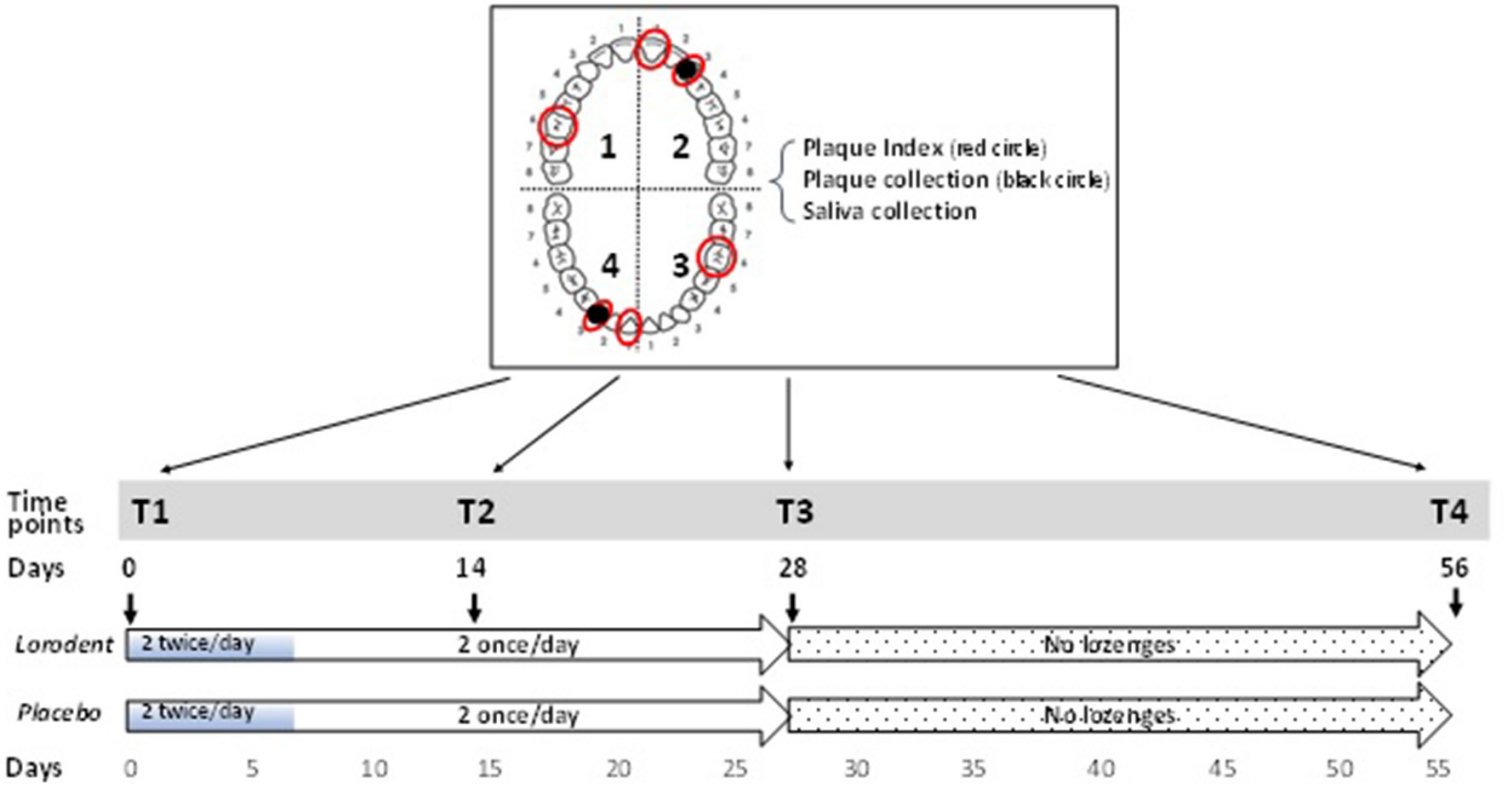
Experimental design and time points of clinical measurements and sample collection. Two lozenges (Lorodent or placebo) were taken twice a day for 7 days (total of 4/day for 7 days = 28 lozenges), followed by 2 lozenges a day for the next 21 days (total of 2/day for 21 days = 42 lozenges); total number of lozenges in trial = 28 + 42. Plaque Index was measured and plaque and saliva samples collected at T1 (0 days), T2 (14 days), T3 (28 days), and T4 (56 days) of the study. The plaque index was measured on teeth # 16, 21, 23, 36, 41, and 43 (red circles) and plaque were collected teeth # 23 and 43 (black circles in tooth arch diagram on top left).

The lozenges were administered for 28 consecutive days, followed by another 28-day follow-up without lozenge administration, for a total trial length of 56 days (Figure 1). Previous studies on probiotic lozenges have examined their use with an ~1-month period of intervention [25, 26]. An additional time point midway through the 28-day intervention was added to the present study in attempt to gain a better understanding of any progression of changes that may occur. A fourth time point was added 28 days after cessation of lozenge administration to evaluate if any potential changes would persist after discontinuation of lozenge administration. An initial loading dose of two lozenges two times per day (between 7:00–9:00 a.m. and 7:00–9:00 p.m.) for the first 7 days, followed by a maintenance dose of two lozenges once a day (between 7:00 and 9:00 a.m.) for the next 21 days was prescribed. The total administration period was 28 days, in line with other similar studies [25, 26] and previous safety assessments with *S. salivarius* K12 (1 × 10^10^ CFU) [27]. Participants of both groups were given standardized oral hygiene instructions and information on how and when to take the lozenges, based on a written script used by both the examiners (FE and SH who were not involved in the orthodontic treatment of the participants and who met to calibrate each other prior to the start of patient contact). Specifically, subjects were instructed to take the lozenges after their tooth brushing and to slowly dissolve the lozenges on their tongue for 5 min without chewing or swallowing and not to brush or rinse their mouth for 1 h following administration of the lozenges. Subjects were also told, based on the written script, to maintain the current standard of care regarding oral hygiene, i.e., to brush two times per day and floss once per day at a minimum. They were also instructed to not brush their teeth before the appointment or upon arrival to the clinic or to use any antimicrobial mouth rinse during the 56-day trial period. Any adverse events at each appointment or immediately after ingestion, e.g., fever or gastrointestinal discomfort including nausea, vomiting, bloody diarrhea or severe abdominal pain, were noted. Changes to their medical history were also noted throughout course of study.

Data and sample collection were taken at the following four time points (Figure 1):

- T1—baseline examination and sample collection at day 0 and initiation of lozenge administration.
- T2—examination and sample collection at day 14.
- T3—examination and sample collection at day 28. Lozenge administration ceased and all remaining lozenges returned to investigators.
- T4—follow up examination and sample collection at day 56.

Sample collection and clinical measurements at T1, T3, and T4 occurred and coincided with the subject's regular orthodontic visits at the Graduate Orthodontic Clinic. One additional appointment (T2), 14 days after the initial data collection, did not coincide with orthodontic visits. Patients were compensated financially in the form of gift cards for their participation in this clinical trial.

### Outcome Measures

#### Clinical Evaluation of Plaque

The Plaque Index (PI) was used to clinically grade the extent and severity of plaque accumulation [24]. PI scores range from 0 (no plaque in gingival area), 1 (a film of plaque), 2 (moderate) to 3 (abundance of soft matter within the gingival pocket and/or on the gingival margin and adjacent tooth surface) (Supplementary Table S1). A modification was made in the study to the Ramfjord teeth to replace the first premolars with the canines, since the canines have one of the highest incidences of white spot lesion formation during orthodontic treatment [28, 29]. Also, by including subjects with extracted first premolars (a common orthodontic treatment plan and found in over a third of the patients treated in the orthodontic clinic), subjects were recruited from a larger pool. Scores of 0–3 (PI) were assigned for the buccal, lingual, mesial and distal surfaces of teeth # 16, 21, 23, 36, 41, and 43 at four time points (T1, T2, T3, and T4) (Figure 1). A total of 24 (6 teeth with 4 surfaces) PI scores each were documented for each subject at each time point. Each participant's overall plaque status at each time point was represented by composite PI (cPI) scores, obtained by adding all 24 PI scores. PI was assessed by two calibrated examiners (FE and SH). Alignment and assessment of examiner scoring were performed at the start of the study (Supplementary Material—Methods and Materials).

#### *S. mutans* DNA Quantitation and Real Time Quantitative PCR

Plaque and salivary samples were collected at each time point and analyzed for the levels of *S. mutans* DNA levels. Well established and validated protocols in DNA extraction and real time qPCR using *S. mutans* specific primers (details provided in Supplementary Material—Methods and Materials) were used to quantify the levels of *S. mutans* DNA levels in saliva and plaque.

#### Measurement of Compliance and Lozenge Satisfaction

At each appointment, subjects were queried as to their ability to follow the oral hygiene, use of mouth rinses, etc. Participants recorded days of lozenge intake on a compliance paper calendar. Compliance was assessed based on the percentage of boxes/lozenges from the total number of lozenges of 70. Monitoring of lozenge safety was conducted through verbal questioning at each appointment. At the conclusion of the study, each participant was asked to complete a 10-item “End of Study Questionnaire”, adapted from a similar questionnaire used by the Xylitol for Adult Caries Trial [30] (Supplementary Table S2). The questions in the questionnaire included their satisfaction with taste of lozenges, success at taking 2 lozenges per day for 28 days, difficulty in taking 2 lozenges per day, difficulty in remembering to take the lozenges every day, whether the study length was too long, whether participants feel the need to prevent white spots, decay or gum disease, whether they lost interest in the study, what they think of the effectiveness of the lozenges, the type of lozenges participants believed they were taking and the likelihood of participants using lozenges if they were shown to be effective at reducing white spots, decay or gum disease.

### Statistical Methods

Inter- and intra-rater reliabilities for PI assessments for both FE and SH were computed using weighted kappa statistics and outcomes were interpreted according to Landis and Koch [31]. An independent *T*-test was used to test age differences between groups. Compliance with lozenge intake was appraised at T2 and T3 using a 2-sided chi-square test. The sex distribution in both groups was tested with a chi-square test.

PI scores were considered scalar values as in previous studies (e.g., [32]). A mixed-effect model was used to test between group and within group differences in PI scores using the study group, the timepoint, and the interaction Group-by-timepoint as fixed factors. Wilcoxon Signed Rank tests were used to test within group changes in microbial DNA (from T1 to T3). Mann-Whitney *U*-tests were used to test between-group differences the microbial DNA at each time point. *Post-hoc* comparisons were adjusted using the Bonferroni method. The operator involved in the statistical analysis (IC) was blinded to the allocation of participants to the two groups.

An a priori power analysis was conducted using G^*^Power (Heinrich-Heine-Universität Düsseldorf, Germany) [33]. As this study was not designed to test sex differences, sex was not considered while computing the sample size. A total sample of 49 participants was required to achieve a power of 0.80 using a medium effect size (*d* = 0.5) and an alpha of 0.05 (considering two study groups, 4 timepoints, and interactions). The level of significance was set at *p* < 0.05. SPSS ver. 24 (IBM Corp. Released 2016. IBM SPSS Statistics for Windows, Armonk, NY: IBM Corp.) was used for the statistical analyses.

## Results

### Subject Recruitment Demographics

**Figure 2:**
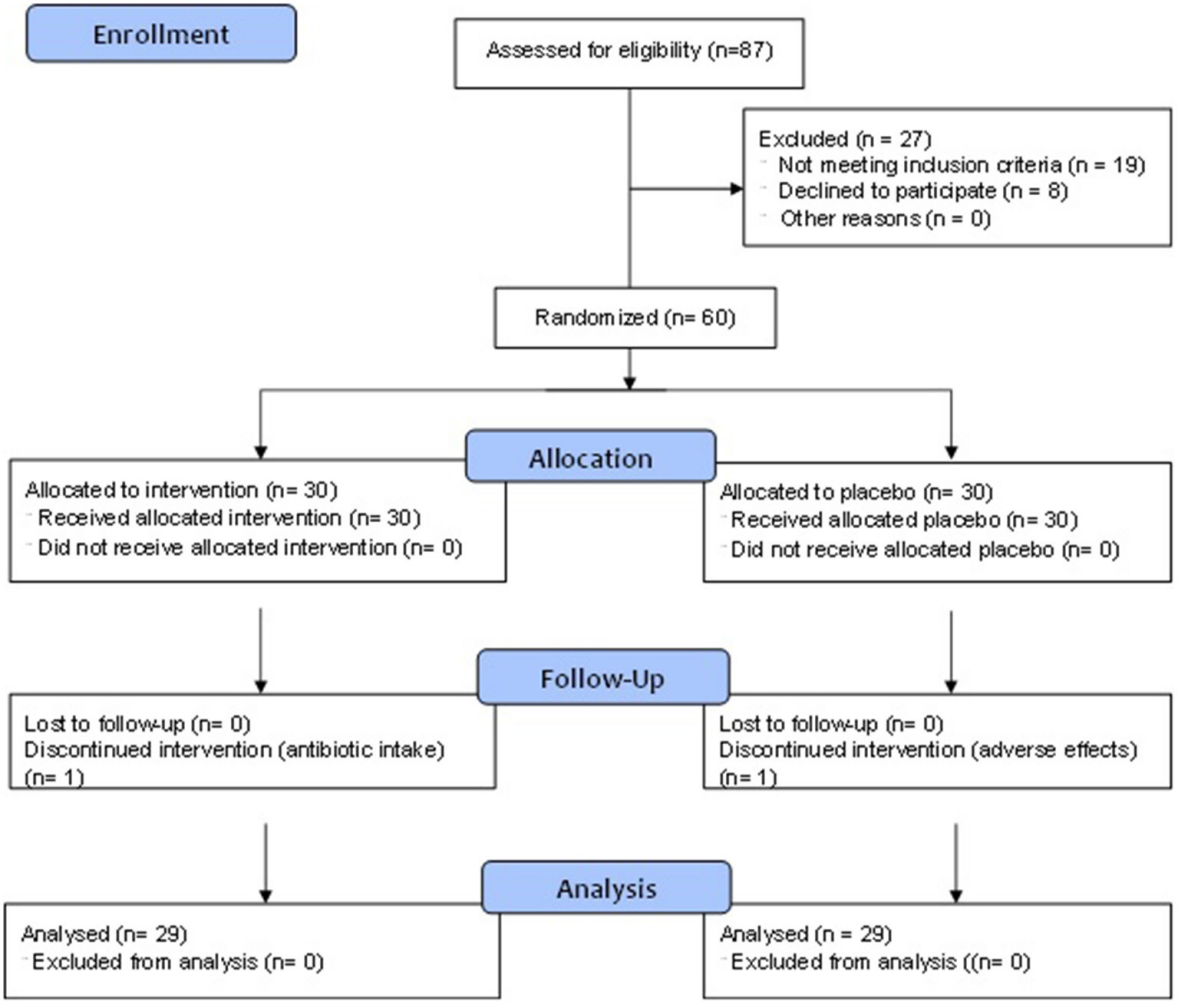
CONSORT flow diagram.

Out of the 87 subjects screened, 60 met the eligibility criteria and were randomized into two equal groups of 30 each in the probiotic/placebo lozenge groups (Figure 2). A final number of 29 subjects in each in the probiotic/placebo groups was obtained—one participant from the probiotic group withdrew due to antibiotic intake following an accident and a participant from the placebo group was withdrawn due to reports of adverse events (gastrointestinal discomfort and diarrhea) after initiation of study.

Demographic characteristics did not significantly differ between the two groups. Overall, more females were enrolled in the study compared to males (56.9% females, 43.1% males)—in the probiotic group, 16/29 (55.2%) were males and 13/29 (44.8%) were females compared to 9/29 (31.0%) males, and 20/29 (69.0%) females in the placebo groups. The distribution of male and female participants did not differ significantly across groups [(*X*^2^ = 1, *N* = 58) = 3.445, *p* = 0.063]. The mean ± SD participants' ages were 15.7 ± 1.7 years; the mean ± SD age in the probiotic group was 15.75 ± 1.67 years compared to 15.64 ± 1.75 years in the placebo group. The groups were similar in age (*p* = 0.807).

### Rater Reliability

Examiner alignment before the study resulted in “substantial” agreement between the examiners for Samples B (intra-oral photos: kappa = 0.72 for PI, *p*-values for both of < 0.001) and C (live clinical patients: kappa = 0.74 for PI; *p*-values for both of < 0.001). After the study, the inter-rater agreement improved to “almost perfect” (kappa = 0.82 for PI; *p*-values for both of < 0.001) when the examiners re-scored Sample B. Intra-rater reliability of the PI scores assigned to Sample B (intraoral photos) showed that both examiners independently had “almost perfect” agreement (PI- examiner 1: kappa = 0.83, examiner 2: kappa = 0.84; *p*-values < 0.001). The intra-rater reliability was not significantly different between examiners (*p* < 0.001).

### Compliance With Intervention, Patients' Satisfaction, and Adverse Events

Analysis of compliance with lozenge intake at T2 and T3 revealed that all participants reported compliance of over 90%. The mean values between groups were also very similar: At T2 and T3, 89.7% (*p* = 1.00) and 72.4% (*p* = 0.56) of subjects in both groups reported a perfect compliance, respectively, with no significant difference between the two groups.

The lozenges were well received by subjects, with 89.6% of participants reportedly very satisfied or satisfied with the lozenge taste and 81% responded they would be fairly likely to use them if they were shown to be effective at reducing white spots, decay or gum disease.

One participant, later identified to be in the placebo group, discontinued use of lozenges 2 weeks after the initial intake of lozenges due to reports of gastrointestinal pain and diarrhea that continued for a couple of days after discontinuation of lozenge administration. The subject was monitored for another 6 weeks, with no recurrence of symptoms. None of the participants in the probiotic group reported any adverse events.

### Effects of the Interventions on Plaque Index and Bacterial Levels

**Figure 3:**
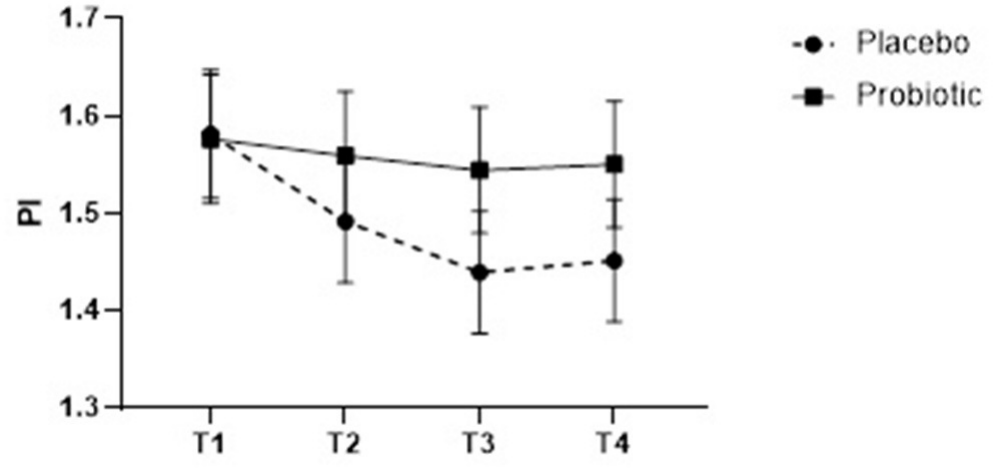
Plaque Index (PI) scores of participants taking control and probiotic lozenges at the 4 time points of the study. Note that the PI scores range from 1 to 4 (Supplementary Table S1) and PI scores shown in both groups ranged between 1.4 and 1.6.

PI scores were not different between groups (*F* = 0.866, *p* = 0.347) or timepoints (Group-by-timepoint interaction *F* = 0.629, *p* = 0.596). No significant improvements in both PI and cPI scores from baseline were seen throughout the intervention period for the probiotic group at any time frame (*p* > 0.05) (Figure 3, show plots depicting temporal changes of PI in both groups).

**Figure 4:**
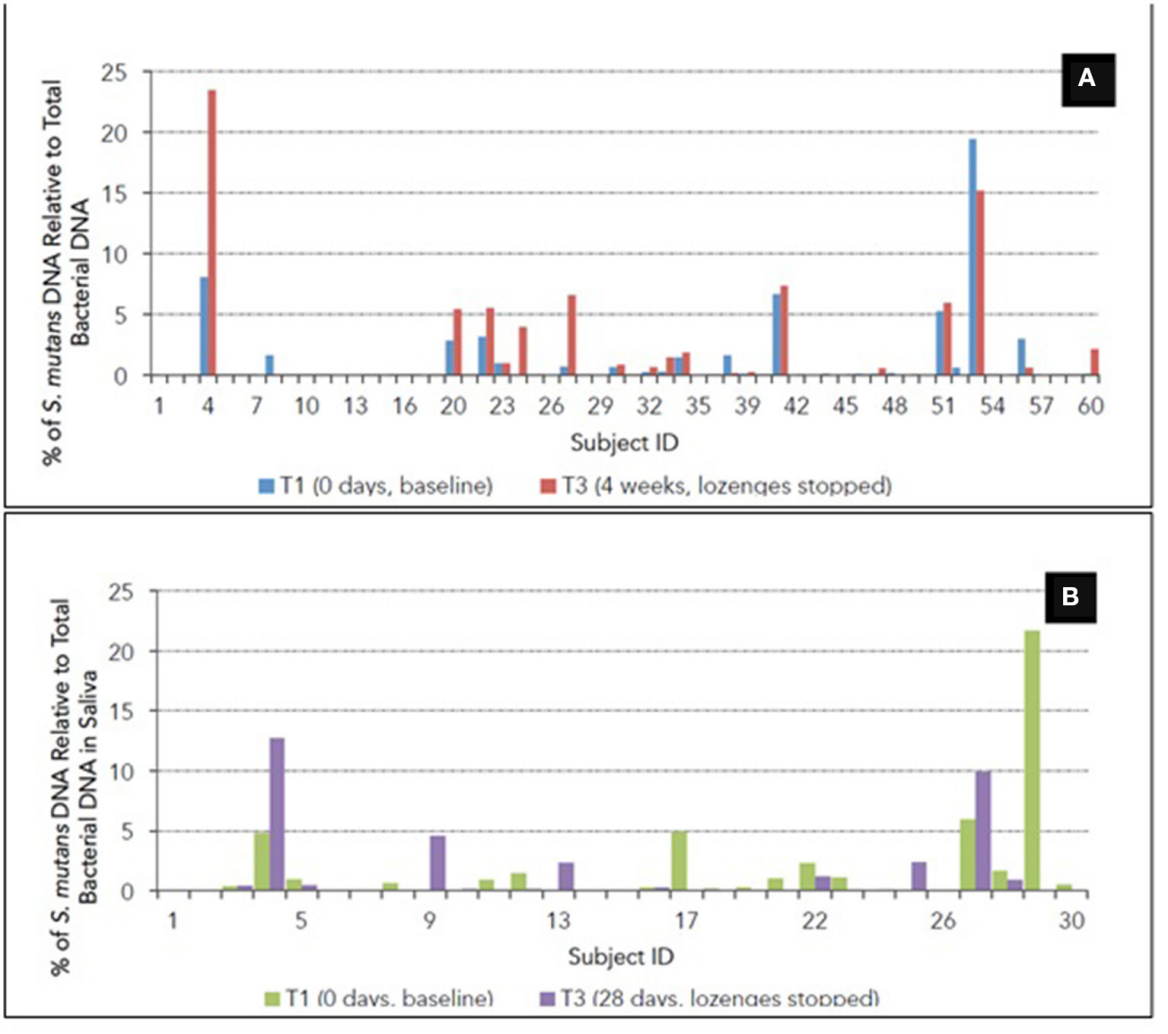
Percentage of *S. mutans* DNA relative to total bacterial DNA from **(A)** supragingival plaque and **(B)** saliva for each participant at T1 and T3. In the majority of samples, *S. mutans* DNA levels were not detectable.

Supragingival plaque and salivary samples were analyzed from 58 (29 probiotic; 29 placebo) and 29 (15 probiotic; 14 placebo) at T1 and T3 for the relative levels of *S. mutans* DNA. The average DNA yields from the plaque and saliva samples were 1.2 and 5.5 ng/μL, respectively. In general, the relative proportions of *S. mutans* DNA in the plaque and saliva samples were relatively low and undetectable in 20 plaque and 3 saliva samples (Figure 4). Of the remaining samples with detectable values, no significant differences in the amount of change in the relative proportions of *S. mutans* were found between the two groups (Table 1). A trend, however, for the *S. mutans* levels to decrease (*p* = 0.372), and increase (*p* = 247), was noticed in the supragingival plaques in the probiotic and placebo groups, respectively.

**Table 1:** Changes in *S. mutans* DNA in plaque and saliva.

**Sample**	**T1 median (IQR)**	**T3 median (IQR)**	**^**§**^*P*-value**
**Probiotic**			
% *S. mutans* DNA in supragingival plaque (*n* = 18)	0.0501 (0.224)	0.039 (0.561)	0.372
% *S. mutans* DNA in saliva (*n* = 14)	0.116 (0.965)	0.259 (2.039)	0.875
**Placebo**			
% *S. mutans* DNA in supragingival plaque (*n* = 20)	0.208 (1.900)	0.584 (2.585)	0.247
% *S. mutans* DNA in saliva (*n* = 12)	0.774 (1.303)	0.0773 (0.132)	0.117

*§ Wilcoxon Signed Ranked test*.

## Discussion

This current study joins the many previous studies designed to better understand the efficacy of a probiotic strategy in the improvement of oral health. The vulnerability of orthodontic patients to caries and poor gingival health and the built-in nature of recurrent orthodontic visits over an extended period make them especially suitable as study subjects for a probiotic study. Additionally, any findings generated in this population group are applicable to the understanding of the effectiveness of oral probiotics in improving oral health in all individuals, whether under orthodontic treatment or not.

Our findings of a lack of statistical differences in PI and salivary and plaque *S. mutans* levels between the probiotic to control groups were corroborated by other studies targeting the orthodontic population. For example, Benic et al. [11] and Kohar et al. [34] found no statistically significant differences in PI measures in orthodontic subjects given *Limosilactobacillus reuteri* (formerly *L. reuteri*) and *S. salivarius* M18 lozenges, respectively. In addition, Gizani et al. [17] did not find a statistical significant difference in salivary MS counts in subjects consuming lozenges containing two strains of *Limosilactobacillus reuteri* once daily for 17 months. In contrast, a study found that the proportion of streptococci was significantly reduced, compared to the administered probiotic strains containing combinations of *Enterococcus* and *Lactobacillus* strains [35]. Also, the authors of a systematic review concluded that, of the 9 included randomized controlled trials, 8 provided evidence that probiotics “improves oral health in patients undergoing fixed orthodontic therapy” [22]. Interestingly, several of these studies used yogurt as a delivery vehicle, suggesting that dairy products might be more effective carrier vehicles for probiotics for oral health, rather than lozenges, a point reinforced by a systematic review and meta-analysis that concluded the effectiveness of dairy products in reducing *S. mutans* levels [36]. Indeed, bacteria such as *Lacticaseibacillus rhamnosus* (formerly *L. rhamnosus*) [37], *Lacticaseibacillus rhamnosus* with *Bifidobacterium animalis* subsp. *lactis* [38], and *S. salivarius* M18 [1] appeared to be effective in reducing dental caries, especially if milk is used as a delivery vehicle (reviewed in [36]). Because dairy products contain calcium phosphate and casein phosphopeptides, enamel remineralization of the carious tooth can be enhanced [39], especially when these probiotics are supplemented with fluoride, another product known to improve remineralization [40]. Although the Lorodent Probiotic Complex was formulated with the premise that a combination of bacterial strains might have a synergistic effect that together would be effective in combating oral diseases, similar to some probiotics used for gastrointestinal health [41, 42], that expectation of synergistic effect with combined probiotic strains was not achieved in the current study. One potential reason for the lack of any clinically measurable effects of the probiotic complex on plaque accumulation could be due to the fact that the active CFUs/lozenge was ~10^5^ CFUs/lozenge, an amount below the 10^9^ active CFUs/lozenge usually used for probiotic therapy in the gastrointestinal literature [1, 38, 40, 43]. Future studies could be dedicated toward using dairy products to carry the Lorodent Complex, a higher dose of the probiotic complex in addition to the adoption of evaluation of its anti-caries properties, e.g., measurement of the presence of white spot lesions, and changes in microbial composition of the plaque and/or saliva during administration of the probiotic (e.g., by next-generation sequencing).

Although no differences were detected in the PI scores or microbiological measurements of *S. mutans* in supragingival plaque or saliva in subjects taking the probiotic complex, one finding from the study stood out clearly. That is, the current study showed a strikingly high compliance of 90% with the blueberry-flavored lozenge use, even when it involved the necessity of taking the lozenges twice a day for 7 days. Many past studies of prescribed drug regimens in adolescence showed compliance rates around 50% (reviewed in [44]). The high compliance and acceptability of these lozenges suggest that commercialization marketing of lozenges as a delivery vehicle for anti-caries probiotics or any therapeutic modality will be well received by adolescents, a major target group of anti-caries efforts. Additionally, no adverse events were experienced in the probiotic group in this study, corroborating the safety shown in other preclinical studies of the probiotic species included in the Lorodent lozenge [27], and thereby establishing the safety of the Lorodent Probiotic Complex in humans at the dosage tested.

Dental crowding has been directly associated with plaque accumulation [45]. In this study, only subjects that had been treated orthodontically for longer than 6 months, with the majority of the subjects being treated longer than 9 months, where little or no crowding were observed. The lack of crowding and the regular monitoring of their oral hygiene suggest that factors related to the probiotics might be at play in the outcome of the study. For example, it is also possible that a longer probiotic treatment may be needed to establish the Lorodent probiotic strains in the oral cavity—oral hygiene in adolescent orthodontic patients is usually poor and mechanical debridement around the orthodontic bracket more arduous, resulting in oral biofilms that are thicker, denser and generally more pathogenic. Indeed, previous studies have also shown that *S. mutans* levels increased about four times in patients undergoing active orthodontic treatment compared to controls [46]. In this regard, perhaps it may help to have complete mouth disinfection with chlorhexidine prior to probiotic usage to increase the ability of the probiotic strains to compete and establish themselves, as had been previously reported [47].

In the current study, we measured the levels of *S. mutans* DNA in the saliva and plaque in addition to the PI scores, both of which are indicators of oral health. Although plaque accumulation itself is not a risk factor for caries, the dental biofilm is a critical component involved in the development of caries and periodontal diseases, both of which are the leading causes of tooth loss [48]. We observed that there was no significant difference in the PI scores in both probiotic and control groups. The Silness and Löe's Plaque Index, as used in the current study, may suffer from lack of accuracy and reliability for the current study. The index is used in periodontology to assess plaque in marginal gingival areas; in contrast, plaque in orthodontic patients usually accumulates in the direct vicinity of the bracket. Furthermore, the scale may not be precise enough to detect less noticeable changes in plaque levels produced by the probiotic. Although alternative plaque indexing scales exist, e.g., the Orthodontic Plaque Index (OPI) [49], or the use of a digital plaque image analysis (DIPA; using a digital camera, UV flash units and software evaluation) [50], they suffer from lack of validation and/or are expensive and technically demanding. In the microbial analysis, although we used a highly sensitive and specific technique (real-time quantitative PCR), the vast number of samples in the current study necessitated the use of a multiplex DNA extraction kit that, although convenient, tends to result in lower yields of DNA that in turn might have resulted in lack of detection of *S. mutans* in many of the plaque samples. Finally, it must be noted that this study was based on an *a priori* sample size calculation for which we considered a medium effect size, and not on data retrieved from other studies or pilot investigations using a similar research design or methods. Computing the sample size using data from previous studies or pilot investigations could have improved the quality of our sample size calculation. Also, our study was not designed and sufficiently powered to test the effect of age and sex on the outcome measures. However, the study groups in the current study had similar age and sex distributions.

Our study illustrated that probiotic therapy in the forms of lozenges at the specific formulation and bacterial count did not influence the plaque accumulation and *S. mutans* levels in saliva and supragingival plaque over the 2-month period in patients undergoing orthodontic treatment. Our study also showed that probiotic lozenges can be practically implemented in the clinical orthodontic setting, in terms of its acceptability by adolescents. Future probiotic studies should focus on bacterial strains, e.g., that of the *S. salivarius* M18 [1], with strong initial *in vitro* evidence to have anti-caries activities, the most effective dosage and frequency of administration for any strains or combination of strains and delivery vehicle. Future probiotic studies could also investigate combination therapy with chlorhexidine or fluoride to increase the probiotic's effectiveness and the possibility of incorporating a pretreatment phase of oral disinfection using chlorhexidine to reduce the oral bacterial load prior to probiotic administration favor probiotic colonization of the oral microbiome.

## Conclusions

The results from this randomized, double-blind, parallel-group, placebo-controlled trial suggested that, although the Lorodent Probiotic Complex was not effective at improving the plaque index or the salivary and plaque levels of *S. mutans* among adolescents undergoing fixed orthodontic appliance therapy, there was high compliance and acceptability of the product. Future studies utilizing probiotics against oral diseases will need to focus on variables such as efficacious and higher dosages of bacterial strains and delivery vehicles, different dosages and frequencies of administration, and possible combinations with chlorhexidine and/or fluoride, and high bacterial enumeration.

## Data Availability Statement

The raw data supporting the conclusions of this article will be made available by the authors, without undue reservation.

## Ethics Statement

The studies involving human participants were reviewed and approved by University of Toronto Human Ethics Board. Written informed consent to participate in this study was provided by the participants' legal guardian/next of kin. Informed consent was obtained from all individual participants included in the study.

## Author Contributions

FE, SM, S-GG, PC, and JB conceived and designed the experiment. FE, SM, KJ, and KM performed the experiment. FE, SM, S-GG, IC, and CL analyzed the data. IC statistical analyses. FE, SM, and S-GG wrote the draft manuscript. All authors reviewed and approved the final manuscript.

## Funding

This work was supported with funds from Ontario Centers for Excellence/Natural Sciences and Engineering Research Council (OCE/NSERC): Lead Investigator—Cadieux PA and Burton JP.

## Conflict of Interest

The authors declare that the research was conducted in the absence of any commercial or financial relationships that could be construed as a potential conflict of interest.

## Publisher's Note

All claims expressed in this article are solely those of the authors and do not necessarily represent those of their affiliated organizations, or those of the publisher, the editors and the reviewers. Any product that may be evaluated in this article, or claim that may be made by its manufacturer, is not guaranteed or endorsed by the publisher.
