# Supplementary material for: Effectiveness of the Lorodent Probiotic Lozenge in Reducing Plaque and Streptococcus mutans Levels in Orthodontic Patients: A Double-Blind Randomized Control Trial
Source: Front Oral Health. 2022 Apr 27;3:884683. doi: 10.3389/froh.2022.884683 (PMC9093136; doi:10.3389/froh.2022.884683)
Supplement: Supplementary file 1 [file Data_Sheet_1.PDF]

Supplementary Materials

Methods and Materials

Randomization *- Sequence generation, allocation concealment mechanism, implementation and blinding*

Upon enrollment, each participant was assigned a unique numerical subject ID number that was used in all subsequent data collection and labeling to maintain confidentiality. Blocked randomization was performed using randomization sequence (random.org) for a predetermined 1:1 allocation ratio to ensure each group was the same size and matched to the subject IDs. One investigator (S-GG) allocated the participants to the study groups.

Recruitment and examination of participant was conducted by SH and FE. Examiners or participants were not informed of the intervention/placebo control grouping. Both probiotic and placebo lozenges looked and tasted the same, with their identities concealed and provided to the examiners with bottles marked either Lozenge A or Lozenge B. Integra Medical Inc. provided the blinded, pre-coded lozenges directly to the investigators but was not involved in any collection or analysis of data. One of the researchers (S-GG) maintained a sealed master list from Integra Medical Inc. of the lozenges' identities. After data analysis was completed, Integra Medical Inc. informed the investigators that Lozenge A was the placebo and Lozenge B was the Lorodent probiotic. These identities were then confirmed by Integra Medical Inc. using bacterial enumeration following their Lorodent Enumeration Protocol.

Examiner alignment and assessment

Examiner alignment and assessment of the two principal examiners (FE and SH) in scoring PI were performed according to Hefti and Preshaw (2012). Initial alignment was accomplished by examination of intra-oral photos from a sample of 5 carefully selected subjects (Sample A) who represented the full range of the scoring system of PI. After examining the buccal, mesial and distal surfaces of teeth #21, 23, 41 & 43, scores were called verbally, site by site; any discrepancies between examiners were discussed. Reproducibility of the examiners' scores was next assessed by scoring the intra-oral photos of another 5 subjects (Sample B) selected to represent the full range of the scoring system. Scoring was next conducted on 3 orthodontic patients (Sample C) of all 4 surfaces (buccal, mesial, distal and lingual) for the 6 teeth (#16, 21, 23, 36, 41 & 43) as described in the protocol of the study. Scores from Samples B and C were recorded to measure the examiners' inter-rater reliability. After study completion, the examiners re-scored the intra-oral photos from Sample B to measure their intra-rater reliability.

S. mutans DNA quantitation and real time quantitative PCR (qPCR)

Both plaque and salivary samples were collected at each time point for microbial analyses. Supragingival plaque were collected with a sterile stainless steel periodontal Gracey curette from the facial surface around the orthodontic brackets of #23 and #43 and combined. Approximately 2 mL of unstimulated saliva was collected by having the participants expectorate in an upright position into a 15 mL Falcon tube. After collection, both plaque and salivary samples were immediately transferred to a -20^{0}C freezer upon collection and processed within two weeks.

DNA was extracted from the plaque and saliva samples using the PowerSoil®-htp 96 Well Soil DNA Isolation Kit (MoBio). Saliva samples were thawed prior to extraction, while the plaque samples were suspended and mixed thoroughly in 400 µL of 1x TE buffer (10 mM Tris [pH 8.0],

1 mM EDTA). Two hundred μL of each were used for DNA extractions according to the manufacturer's protocol with two changes: the addition of a 10-min incubation step at 65°C in a bead bath before the bead-beating step, and a doubling of all centrifugation times. Extracted DNAs were quantified using a NanoDrop 1000 Spectrophotometer (Thermo Scientific) and stored at -20°C until used for qPCR.

Validated primers specific for *S. mutans* (Childers *et al.* 2011): F: $^{5'}$GCCTACAGCTCAGAGATGCTATTCT$^{3'}$; R: $^{5'}$GCCATACACCACTCATGAATTGA$^{3'}$ were tested against isolated DNA from pure cultures of *S. mutans* as a positive control to confirm their efficiency and specificity. Primers to detect total bacteria (Galimanas *et al.* 2014) were also used: F: $^{5'}$TCCTACGGGAGGCAGCAGT$^{3'}$ R: $^{5'}$GGACTACCAGGGTATCTAATCCTGTT$^{3'}$ All qPCR reactions were carried out in 384-well reaction plates using the 7900 HT Sequence Detection System (Applied Biosystems, Foster City, California, USA) under the following program: Stage 1, 50°C for 2 min; Stage 2, 95°C for 10 min; then 95°C for 15 sec, and 60°C for 1 min, for 40 cycles. Reactions were carried out in 20 μL volumes with 5 μL extracted template DNA, 10 uL 1×*Power* SYBR Green PCR Master Mix (Applied Biosystems), 4.5 uL PCR-grade water, and 0.25 μL of each forward and reverse primer (100 μM stock). Standard curves were generated for the primer sets using serial 10-fold dilutions of known concentrations of isolated DNA from pure cultures of *S. mutans*. Bacterial species were quantified in triplicate (technical replicates with the exact same reaction mixture) for each biological sample to control for errors from inaccurate pipetting. The mean cycle threshold (Ct) values of the triplicates were used to determine raw DNA concentrations based on the standard curves. However, since the amount of plaque or saliva per sample was not standardized, the amount of extracted DNA loaded in each qPCR reaction was different for each sample. To control for this and to allow for comparison across samples, the total amount of bacterial DNA was set as an internal reference for each sample, and relative proportion of *S. mutans* DNA was calculated as a percentage of that.

Table S1. Plaque index (PI) scoring criteria [Löe 1967]
0 – No plaque in gingival area
1 – A film of plaque adhering to the free gingival margin and adjacent area of the tooth, which cannot be seen with the naked eye, only seen by running a probe across the tooth surface
2 – Moderate accumulation of plaque deposits within the gingival pocket, on the gingival margin and/or adjacent tooth surface, which can be seen with the naked eye
3 – Abundance of soft matter within the gingival pocket and/or on the gingival margin and adjacent tooth surface

Löe H. 1967. The Gingival Index, the Plaque Index and the Retention Index Systems. J Periodontol 38(6):Suppl:610-616.

.

Table S2. End of Study Questionnaire

Questions	Responses	Results
1. How satisfied were you with the taste of the lozenges in the study?	Very satisfied<br>Satisfied<br>Neutral<br>Dissatisfied<br>Very dissatisfied	89.6% responded very satisfied or satisfied with the lozenge taste, no responses for dissatisfaction
2. During the 28 day administration period of the study, how successful were you taking 2 lozenges per day?	Completely Successful (rarely missed taking a lozenge)<br>Mostly Successful (missed taking a few to several lozenges each week)<br>Somewhat Successful (missed taking about half of my lozenges each week) Mostly Unsuccessful (missed taking most of my lozenges)<br>Completely Unsuccessful (stopped taking the lozenges)	84.5% felt completely successful, 15.5% felt they were most successful, no responses for unsuccessful
3. It was difficult to take 2 lozenges per day.	Strongly agree<br>Agree<br>Neither Disagree<br>Strongly disagree	93.2% disagreed or strongly disagreed, 5.2% were neutral, and 1.7% agreed.
4. Remembering to take the lozenges every day was difficult.	Strongly agree<br>Agree<br>Neither Disagree<br>Strongly disagree	67.3% disagreed or strongly disagreed, 19.0% were neutral and 13.7% agreed.
5. The length of the study (2 months) was too long.	Strongly agree<br>Agree<br>Neither Disagree<br>Strongly disagree	74.1% disagreed or strongly disagreed, 20.7% were neutral, and 5.2% agreed.
6. I did not feel the need to prevent white spots, decay or gum disease.	Strongly agree<br>Agree<br>Neither Disagree<br>Strongly disagree	77.6% disagreed or strongly disagreed, 17.2% neutral, 5.2% agreed.
7. I lost interest in the study.	Strongly agree<br>Agree<br>Neither Disagree<br>Strongly disagree	86.2% disagreed or strongly disagreed, 13.8% were neutral
8. In your opinion, how effective are the lozenges?	Very effective<br>Effective<br>Neutral Ineffective<br>Very ineffective	62% believed the lozenges were effective or very effective, 36.2% were neutral, and 1.7% did not believe they were effective.
9. What type of lozenges do you think you were getting?	Probiotic<br>Placebo	86.2% believed they were taking the probiotic, 13.8% believed they were taking the placebo.
10. If the lozenges were shown to be effective in reducing white spots and gum disease, how likely would you be to use the lozenges in the future?	Very likely<br>Moderately likely<br>Somewhat likely<br>Somewhat not likely<br>Very unlikely	81% responded they would be very likely or moderately likely to use them, 15.5% were somewhat likely, and 3.4% were somewhat not likely.

References

Childers, N.K., Osgood, R.C., Hsu, K.L., Manmontri, C., Momeni, S.S., Mahtani, H.K., Cutter, G.R. and Ruby, J.D. (2011) 'Real-time quantitative polymerase chain reaction for enumeration of Streptococcus mutans from oral samples', *Eur J Oral Sci*, 119(6), 447-54, available: http://dx.doi.org/10.1111/j.1600-0722.2011.00888.x.

Galimanas, V., Hall, M.W., Singh, N., Lynch, M.D., Goldberg, M., Tenenbaum, H., Cvitkovitch, D.G., Neufeld, J.D. and Senadheera, D.B. (2014) 'Bacterial community composition of chronic periodontitis and novel oral sampling sites for detecting disease indicators', *Microbiome*, 2, 32, available: http://dx.doi.org/10.1186/2049-2618-2-32.

Hefti, A.F. and Preshaw, P.M. (2012) 'Examiner alignment and assessment in clinical periodontal research', *Periodontol 2000*, 59(1), 41-60, available: http://dx.doi.org/10.1111/j.1600-0757.2011.00436.x.
